# 1,4-Bis(4-pyridylmeth­yl)piperazin-1-ium perchlorate fumaric acid hemisolvate

**DOI:** 10.1107/S1600536809026026

**Published:** 2009-07-11

**Authors:** Gregory A. Farnum, Robert L. LaDuca

**Affiliations:** a Lyman Briggs College, Department of Chemistry, Michigan State University, East Lansing, MI 48825, USA

## Abstract

In the title salt, C_16_H_21_N_4_^+^·ClO_4_^−^·0.5C_4_H_4_O_4_, fumaric acid mol­ecules, situated across crystallographic inversion centres, are O—H⋯N hydrogen bonded to two protonated 1,4-bis­(4-pyridylmeth­yl)piperazine cations, forming trimolecular units. These construct one-dimensional supra­molecular ribbons by N—H⋯N hydrogen bonding, and further aggregate *via* π–π inter­actions [shortest C⋯C contact = 3.640 (1) Å] and perchlorate-mediated C—H⋯O inter­actions.

## Related literature

For the preparation of bis­(4-pyridylmeth­yl)piperazine, see: Pocic *et al.* (2005 ▶). For a cadmium fumarate coordination polymer containing bis­(4-pyridylmeth­yl)piperazine, see: Martin *et al.* (2009 ▶).
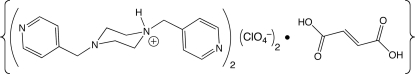


## Experimental

### 

#### Crystal data

C_16_H_21_N_4_^+^·ClO_4_^−^·0.5C_4_H_4_O_4_
*M* *_r_* = 426.85
Triclinic, 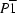

*a* = 7.7287 (9) Å
*b* = 9.6415 (11) Å
*c* = 14.3440 (17) Å
α = 88.691 (2)°
β = 83.785 (2)°
γ = 66.749 (2)°
*V* = 976.0 (2) Å^3^
*Z* = 2
Mo *K*α radiation
μ = 0.24 mm^−1^
*T* = 173 K
0.41 × 0.21 × 0.13 mm

#### Data collection

Bruker APEXII diffractometer
Absorption correction: multi-scan (*SADABS*; Sheldrick, 1996 ▶) *T*_min_ = 0.908, *T*_max_ = 0.969
14571 measured reflections
3593 independent reflections
3178 reflections with *I* > 2σ(*I*)
*R*_int_ = 0.031

#### Refinement

*R*[*F* ^2^ > 2σ(*F* ^2^)] = 0.036
*wR*(*F* ^2^) = 0.092
*S* = 1.05
3593 reflections
268 parameters
H atoms treated by a mixture of independent and constrained refinement
Δρ_max_ = 0.36 e Å^−3^
Δρ_min_ = −0.38 e Å^−3^

### 

Data collection: *APEX2* (Bruker, 2006 ▶); cell refinement: *APEX2*; data reduction: *SAINT* (Bruker, 2006 ▶); program(s) used to solve structure: *SHELXS97* (Sheldrick, 2008 ▶); program(s) used to refine structure: *SHELXL97* (Sheldrick, 2008 ▶); molecular graphics: *Crystal Maker* (Palmer, 2005 ▶); software used to prepare material for publication: *SHELXL97* and *PLATON* (Spek, 2009 ▶).

## Supplementary Material

Crystal structure: contains datablocks I, global. DOI: 10.1107/S1600536809026026/tk2492sup1.cif

Structure factors: contains datablocks I. DOI: 10.1107/S1600536809026026/tk2492Isup2.hkl

Additional supplementary materials:  crystallographic information; 3D view; checkCIF report

## Figures and Tables

**Table 1:** Hydrogen-bond geometry (Å, °)

*D*—H⋯*A*	*D*—H	H⋯*A*	*D*⋯*A*	*D*—H⋯*A*
N3—H3*N*⋯N4^i^	0.925 (19)	1.884 (19)	2.8067 (19)	175.0 (17)
O1—H1*A*⋯N1^ii^	0.93 (2)	1.68 (2)	2.6081 (19)	178 (2)
C10—H10*A*⋯O3^iii^	0.99	2.37	3.281 (2)	153 (2)
C12—H12⋯O6^iv^	0.95	2.49	3.138 (3)	126 (2)

Symmetry codes: (i) 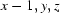; (ii) 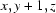; (iii) 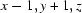; (iv) 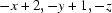.

**Fig. 1.**
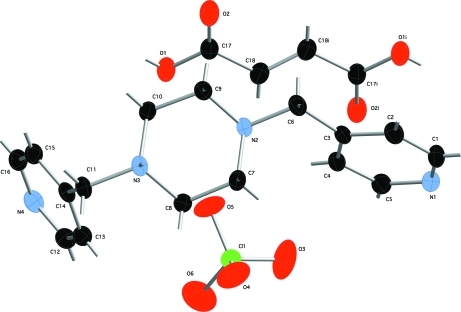
Asymmetric unit of (I), showing 50% probability ellipsoids and atom numbering scheme. Hydrogen atoms positions are marked as gray sticks. A complete fumaric acid moiety is shown. Symmetry code: i -x + 1, -y + 1, -z + 1.

**Fig. 2.**
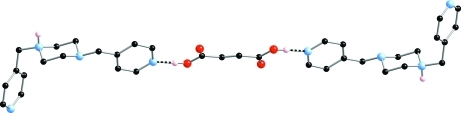
A [(Hbpmp)2(H2fumarate)]2+ trimolecular aggregration in (I). Hydrogen bonding is shown as dashed lines.

**Fig. 3.**
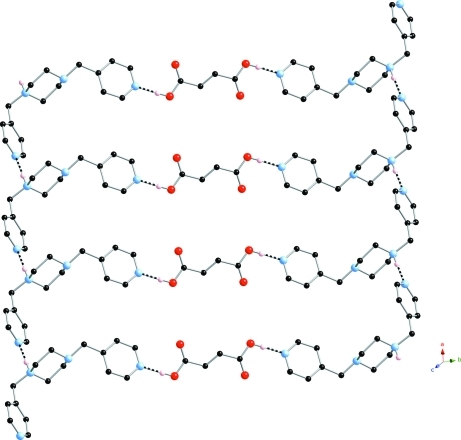
A hydrogen-bonded ribbon motif consisting of [(Hbpmp)2(H2fumarate)]2+ trimolecular aggregration. Hydrogen bonding is shown as dashed lines.

**Fig. 4.**
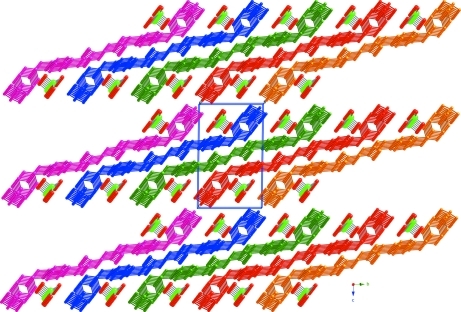
Packing diagram for (I).

## supplementary crystallographic information

### Comment

The title compound (I) was prepared during an attempt to prepare a divalent Cd coordination polymer containing both fumarate and *N,N'*-di(4-pyridyl- methyl)piperazine (bpmp) ligands. The coordination polymer [Cd(fumarate)(bpmp)(H_2_O)_2_]_n_ could only be prepared by using maleic acid, which underwent *in situ cis*-*trans* isomerization (Martin *et al.*, 2009).

The asymmetric unit of the title compound (Fig. 1) consists of one bpmp molecule protonated at one of its piperazinyl-N atoms, one perchlorate anion, and one-half of a fumaric acid molecule situated across a crystallographic inversion centre. Hydrogen-bonding between the carboxylic acid functional groups of the fumaric acid molecules and pyridyl-N atoms within the Hbpmp^+^ moieties produces dicationic [(Hbpmp)_2_(H_2_fumarate)]^2+^ trimolecular aggregations (Fig. 2 & Table 1).

The [(Hbpmp)_2_(H_2_fumarate)]^2+^ units construct one-dimensional ribbon motifs (Fig. 3) by means of N—H···N hydrogen-bonding between the protonated piperazinyl-N atoms and pyridyl-N atoms. Individual ribbons aggregate into a 2-D supramolecular layer through C—H···O interactions mediated by the perchlorate anions (Table 1). Neighbouring layers stack into the 3-D crystal structure (Fig. 4) by π-π interactions between pyridyl rings. (*Cg*—*Cg*(-*x* + 1,-*y* + 2,-*z*) with distance = 3.640 (1) Å).

### Experimental

Cadmium perchlorate hexahydrate and fumaric acid were obtained commercially. *N,N'*-Di(4-pyridylmethyl)piperazine (bpmp) was prepared *via* a published procedure (Pocic *et al.*, 2005). Cadmium perchlorate hexahydrate (0.0175 g, 0.0562 mmol) and fumaric acid (0.0065 g, 0.056 mmol) were placed in water (1.5 ml) in a glass vial along with 1.0 *M* NaOH (0.2 ml). This solution was heated to 373 K to dissolve the fumaric acid. An aliquot (0.75 ml) of a 1:1 water:ethanol solution was carefully layered on top. Then 0.075 *M* ethanolic solution (1.5 ml) of bpmp (0.11 mmol) was carefully layered on top. Colourless blocks of (I) were deposited after standing for one week at 293 K.

### Refinement

All H atoms bound to C atoms were placed in calculated positions, with C—H = 0.95 Å for *sp*^2^ hybridized C atoms and C—H = 0.99 Å for *sp*^3^ hybridized C atoms, and refined in riding mode with *U*_iso_ = 1.2*U*_eq_(C). The H atoms bound to O and the H atoms bound to the piperazinyl-N were found *via* Fourier difference map, and refined with *U*_iso_ = 1.2 times the *U*_eq_(O, N).

### Crystal data

**Table:**

C_16_H_21_N_4_^+^·ClO_4_^−^·0.5C_4_H_4_O_4_	*Z* = 2
*M**_r_* = 426.85	*F*(000) = 448
Triclinic, *P*1	*D*_x_ = 1.452 Mg m^−^^3^
Hall symbol: -P 1	Mo *K*α radiation, λ = 0.71073 Å
*a* = 7.7287 (9) Å	Cell parameters from 14571 reflections
*b* = 9.6415 (11) Å	θ = 2.3–25.4°
*c* = 14.3440 (17) Å	µ = 0.24 mm^−^^1^
α = 88.691 (2)°	*T* = 173 K
β = 83.785 (2)°	Block, colourless
γ = 66.749 (2)°	0.41 × 0.21 × 0.13 mm
*V* = 976.0 (2) Å^3^	

### Data collection

**Table:**

Bruker APEXII diffractometer	3593 independent reflections
Radiation source: fine-focus sealed tube	3178 reflections with *I* > 2σ(*I*)
graphite	*R*_int_ = 0.031
ω–ψ scans	θ_max_ = 25.4°, θ_min_ = 2.3°
Absorption correction: multi-scan (*SADABS*; Sheldrick, 1996)	*h* = −9→9
*T*_min_ = 0.908, *T*_max_ = 0.969	*k* = −11→11
14571 measured reflections	*l* = −17→17

### Refinement

**Table:**

Refinement on *F*^2^	Primary atom site location: structure-invariant direct methods
Least-squares matrix: full	Secondary atom site location: difference Fourier map
*R*[*F*^2^ > 2σ(*F*^2^)] = 0.036	Hydrogen site location: inferred from neighbouring sites
*wR*(*F*^2^) = 0.092	H atoms treated by a mixture of independent and constrained refinement
*S* = 1.05	*w* = 1/[σ^2^(*F*_o_^2^) + (0.0395*P*)^2^ + 0.5777*P*] where *P* = (*F*_o_^2^ + 2*F*_c_^2^)/3
3593 reflections	(Δ/σ)_max_ < 0.001
268 parameters	Δρ_max_ = 0.36 e Å^−^^3^
0 restraints	Δρ_min_ = −0.38 e Å^−^^3^

### Special details

**Table:**

Geometry. All e.s.d.'s (except the e.s.d. in the dihedral angle between two l.s. planes) are estimated using the full covariance matrix. The cell e.s.d.'s are taken into account individually in the estimation of e.s.d.'s in distances, angles and torsion angles; correlations between e.s.d.'s in cell parameters are only used when they are defined by crystal symmetry. An approximate (isotropic) treatment of cell e.s.d.'s is used for estimating e.s.d.'s involving l.s. planes.Supramolecular interactions were calculated using *PLATON* (Spek, 2009).
Refinement. Refinement of *F*^2^ against ALL reflections. The weighted *R*-factor *wR* and goodness of fit *S* are based on *F*^2^, conventional *R*-factors *R* are based on *F*, with *F* set to zero for negative *F*^2^. The threshold expression of *F*^2^ > σ(*F*^2^) is used only for calculating *R*-factors(gt) *etc*. and is not relevant to the choice of reflections for refinement. *R*-factors based on *F*^2^ are statistically about twice as large as those based on *F*, and *R*- factors based on ALL data will be even larger.

### Fractional atomic coordinates and isotropic or equivalent isotropic displacement parameters (Å^2^)

**Table:**

	*x*	*y*	*z*	*U*_iso_*/*U*_eq_	
N2	0.14315 (19)	0.60021 (15)	0.29972 (9)	0.0221 (3)	
N3	0.12885 (19)	0.80520 (15)	0.14915 (9)	0.0195 (3)	
H3N	0.005 (3)	0.828 (2)	0.1385 (13)	0.023*	
C7	0.1416 (2)	0.55727 (19)	0.20264 (12)	0.0251 (4)	
H7A	0.0094	0.5841	0.1892	0.030*	
H7B	0.2108	0.4467	0.1936	0.030*	
N1	0.3877 (2)	0.04563 (16)	0.39116 (10)	0.0283 (3)	
C2	0.0899 (3)	0.2558 (2)	0.39796 (12)	0.0284 (4)	
H2	−0.0442	0.2929	0.4102	0.034*	
C10	0.1253 (2)	0.84885 (18)	0.24896 (11)	0.0226 (3)	
H10A	0.0515	0.9588	0.2586	0.027*	
H10B	0.2561	0.8254	0.2637	0.027*	
C8	0.2342 (2)	0.63786 (18)	0.13586 (12)	0.0241 (4)	
H8A	0.3676	0.6088	0.1480	0.029*	
H8B	0.2333	0.6081	0.0704	0.029*	
C3	0.1772 (2)	0.35304 (18)	0.36903 (11)	0.0234 (4)	
N4	0.76043 (19)	0.86552 (17)	0.10634 (10)	0.0261 (3)	
C11	0.1980 (2)	0.89600 (18)	0.07906 (12)	0.0224 (3)	
H11A	0.1951	0.8615	0.0150	0.027*	
H11B	0.1099	1.0034	0.0862	0.027*	
C9	0.0361 (2)	0.76287 (18)	0.31339 (12)	0.0238 (4)	
H9A	0.0339	0.7917	0.3794	0.029*	
H9B	−0.0962	0.7896	0.3002	0.029*	
C4	0.3730 (2)	0.29139 (19)	0.34948 (11)	0.0244 (4)	
H4	0.4382	0.3534	0.3280	0.029*	
C15	0.4238 (2)	0.99776 (19)	0.13418 (12)	0.0249 (4)	
H15	0.3188	1.0839	0.1600	0.030*	
C16	0.6071 (2)	0.9846 (2)	0.14065 (12)	0.0268 (4)	
H16	0.6248	1.0639	0.1709	0.032*	
C5	0.4719 (3)	0.13881 (19)	0.36167 (12)	0.0273 (4)	
H5	0.6060	0.0981	0.3485	0.033*	
C14	0.3955 (2)	0.88367 (18)	0.08945 (11)	0.0202 (3)	
C6	0.0591 (2)	0.51999 (19)	0.36533 (13)	0.0282 (4)	
H6A	−0.0668	0.5346	0.3472	0.034*	
H6B	0.0398	0.5652	0.4289	0.034*	
C1	0.1992 (3)	0.1050 (2)	0.40882 (12)	0.0300 (4)	
H1	0.1375	0.0403	0.4298	0.036*	
C12	0.7322 (2)	0.7561 (2)	0.06285 (12)	0.0261 (4)	
H12	0.8397	0.6706	0.0383	0.031*	
O1	0.55138 (19)	0.75287 (15)	0.40307 (10)	0.0365 (3)	
H1A	0.496 (3)	0.857 (3)	0.3980 (15)	0.044*	
C17	0.4406 (2)	0.70070 (19)	0.45469 (12)	0.0261 (4)	
Cl1	0.78159 (6)	0.33434 (5)	0.16421 (3)	0.02773 (13)	
O5	0.7247 (2)	0.45702 (18)	0.22864 (11)	0.0539 (4)	
O6	0.9359 (3)	0.3305 (3)	0.10017 (15)	0.0877 (7)	
C18	0.5308 (2)	0.53460 (19)	0.46587 (13)	0.0281 (4)	
H18	0.6356	0.4764	0.4228	0.034*	
O2	0.28278 (17)	0.77780 (14)	0.49090 (10)	0.0342 (3)	
O3	0.8334 (3)	0.19613 (18)	0.21367 (14)	0.0673 (5)	
O4	0.6259 (2)	0.34683 (18)	0.11388 (11)	0.0555 (4)	
C13	0.5544 (2)	0.76136 (19)	0.05197 (12)	0.0243 (4)	
H13	0.5408	0.6823	0.0192	0.029*	

### Atomic displacement parameters (Å^2^)

**Table:**

	*U*^11^	*U*^22^	*U*^33^	*U*^12^	*U*^13^	*U*^23^
N2	0.0246 (7)	0.0196 (7)	0.0218 (7)	−0.0091 (6)	−0.0001 (6)	0.0040 (5)
N3	0.0152 (6)	0.0221 (7)	0.0233 (7)	−0.0091 (6)	−0.0048 (5)	0.0053 (5)
C7	0.0299 (9)	0.0217 (8)	0.0265 (9)	−0.0128 (7)	−0.0051 (7)	0.0032 (7)
N1	0.0347 (8)	0.0221 (7)	0.0273 (8)	−0.0110 (6)	−0.0015 (6)	0.0027 (6)
C2	0.0277 (9)	0.0285 (9)	0.0300 (9)	−0.0131 (7)	0.0002 (7)	0.0046 (7)
C10	0.0253 (8)	0.0197 (8)	0.0240 (8)	−0.0097 (7)	−0.0050 (7)	0.0023 (6)
C8	0.0258 (9)	0.0217 (8)	0.0236 (8)	−0.0083 (7)	−0.0022 (7)	0.0011 (6)
C3	0.0288 (9)	0.0235 (8)	0.0187 (8)	−0.0118 (7)	−0.0010 (7)	0.0033 (6)
N4	0.0213 (7)	0.0348 (8)	0.0265 (7)	−0.0152 (6)	−0.0046 (6)	0.0054 (6)
C11	0.0182 (8)	0.0253 (8)	0.0252 (8)	−0.0097 (7)	−0.0055 (6)	0.0093 (7)
C9	0.0233 (8)	0.0220 (8)	0.0242 (8)	−0.0077 (7)	0.0001 (7)	0.0022 (7)
C4	0.0286 (9)	0.0251 (9)	0.0229 (8)	−0.0147 (7)	−0.0011 (7)	0.0020 (7)
C15	0.0222 (8)	0.0239 (8)	0.0288 (9)	−0.0102 (7)	0.0002 (7)	0.0022 (7)
C16	0.0285 (9)	0.0305 (9)	0.0277 (9)	−0.0185 (8)	−0.0032 (7)	0.0021 (7)
C5	0.0270 (9)	0.0264 (9)	0.0277 (9)	−0.0101 (7)	−0.0019 (7)	0.0014 (7)
C14	0.0184 (8)	0.0242 (8)	0.0201 (8)	−0.0105 (7)	−0.0044 (6)	0.0084 (6)
C6	0.0271 (9)	0.0245 (9)	0.0298 (9)	−0.0091 (7)	0.0051 (7)	0.0054 (7)
C1	0.0371 (10)	0.0262 (9)	0.0317 (9)	−0.0183 (8)	−0.0017 (8)	0.0048 (7)
C12	0.0195 (8)	0.0299 (9)	0.0273 (9)	−0.0088 (7)	−0.0007 (7)	0.0019 (7)
O1	0.0351 (7)	0.0220 (7)	0.0471 (8)	−0.0093 (6)	0.0085 (6)	0.0065 (6)
C17	0.0285 (9)	0.0253 (9)	0.0269 (9)	−0.0127 (7)	−0.0054 (7)	0.0042 (7)
Cl1	0.0276 (2)	0.0264 (2)	0.0286 (2)	−0.01057 (17)	0.00035 (17)	−0.00359 (17)
O5	0.0618 (10)	0.0491 (9)	0.0518 (9)	−0.0238 (8)	0.0021 (8)	−0.0249 (7)
O6	0.0777 (14)	0.1239 (18)	0.0735 (13)	−0.0640 (14)	0.0431 (11)	−0.0279 (12)
C18	0.0270 (9)	0.0237 (9)	0.0335 (9)	−0.0101 (7)	−0.0030 (7)	0.0012 (7)
O2	0.0263 (7)	0.0255 (7)	0.0473 (8)	−0.0082 (5)	0.0007 (6)	0.0072 (6)
O3	0.0694 (12)	0.0396 (9)	0.0948 (14)	−0.0176 (8)	−0.0373 (10)	0.0257 (9)
O4	0.0474 (9)	0.0555 (10)	0.0560 (10)	−0.0064 (8)	−0.0259 (8)	−0.0122 (8)
C13	0.0228 (8)	0.0276 (9)	0.0252 (9)	−0.0127 (7)	−0.0032 (7)	0.0011 (7)

### Geometric parameters (Å, °)

**Table:**

N2—C6	1.462 (2)	C9—H9A	0.9900
N2—C9	1.462 (2)	C9—H9B	0.9900
N2—C7	1.464 (2)	C4—C5	1.382 (2)
N3—C10	1.496 (2)	C4—H4	0.9500
N3—C8	1.499 (2)	C15—C16	1.385 (2)
N3—C11	1.5082 (19)	C15—C14	1.386 (2)
N3—H3N	0.925 (19)	C15—H15	0.9500
C7—C8	1.513 (2)	C16—H16	0.9500
C7—H7A	0.9900	C5—H5	0.9500
C7—H7B	0.9900	C14—C13	1.389 (2)
N1—C1	1.336 (2)	C6—H6A	0.9900
N1—C5	1.340 (2)	C6—H6B	0.9900
C2—C1	1.379 (2)	C1—H1	0.9500
C2—C3	1.388 (2)	C12—C13	1.381 (2)
C2—H2	0.9500	C12—H12	0.9500
C10—C9	1.515 (2)	O1—C17	1.312 (2)
C10—H10A	0.9900	O1—H1A	0.93 (2)
C10—H10B	0.9900	C17—O2	1.214 (2)
C8—H8A	0.9900	C17—C18	1.486 (2)
C8—H8B	0.9900	Cl1—O6	1.4129 (17)
C3—C4	1.388 (2)	Cl1—O5	1.4135 (14)
C3—C6	1.508 (2)	Cl1—O3	1.4271 (16)
N4—C16	1.338 (2)	Cl1—O4	1.4329 (15)
N4—C12	1.339 (2)	C18—C18^i^	1.323 (4)
C11—C14	1.507 (2)	C18—H18	0.9500
C11—H11A	0.9900	C13—H13	0.9500
C11—H11B	0.9900		
			
C6—N2—C9	109.37 (13)	C10—C9—H9B	109.6
C6—N2—C7	110.81 (13)	H9A—C9—H9B	108.1
C9—N2—C7	109.43 (13)	C5—C4—C3	119.24 (15)
C10—N3—C8	109.52 (12)	C5—C4—H4	120.4
C10—N3—C11	113.55 (12)	C3—C4—H4	120.4
C8—N3—C11	113.77 (13)	C16—C15—C14	119.15 (16)
C10—N3—H3N	107.0 (11)	C16—C15—H15	120.4
C8—N3—H3N	107.2 (11)	C14—C15—H15	120.4
C11—N3—H3N	105.2 (11)	N4—C16—C15	123.15 (16)
N2—C7—C8	110.08 (13)	N4—C16—H16	118.4
N2—C7—H7A	109.6	C15—C16—H16	118.4
C8—C7—H7A	109.6	N1—C5—C4	123.06 (16)
N2—C7—H7B	109.6	N1—C5—H5	118.5
C8—C7—H7B	109.6	C4—C5—H5	118.5
H7A—C7—H7B	108.2	C15—C14—C13	117.85 (15)
C1—N1—C5	117.52 (15)	C15—C14—C11	120.58 (15)
C1—C2—C3	119.45 (17)	C13—C14—C11	121.53 (15)
C1—C2—H2	120.3	N2—C6—C3	113.82 (14)
C3—C2—H2	120.3	N2—C6—H6A	108.8
N3—C10—C9	109.59 (13)	C3—C6—H6A	108.8
N3—C10—H10A	109.8	N2—C6—H6B	108.8
C9—C10—H10A	109.8	C3—C6—H6B	108.8
N3—C10—H10B	109.8	H6A—C6—H6B	107.7
C9—C10—H10B	109.8	N1—C1—C2	123.08 (16)
H10A—C10—H10B	108.2	N1—C1—H1	118.5
N3—C8—C7	109.62 (13)	C2—C1—H1	118.5
N3—C8—H8A	109.7	N4—C12—C13	123.18 (16)
C7—C8—H8A	109.7	N4—C12—H12	118.4
N3—C8—H8B	109.7	C13—C12—H12	118.4
C7—C8—H8B	109.7	C17—O1—H1A	112.1 (14)
H8A—C8—H8B	108.2	O2—C17—O1	124.70 (16)
C4—C3—C2	117.62 (16)	O2—C17—C18	122.91 (16)
C4—C3—C6	122.93 (15)	O1—C17—C18	112.39 (15)
C2—C3—C6	119.36 (15)	O6—Cl1—O5	111.03 (11)
C16—N4—C12	117.42 (14)	O6—Cl1—O3	109.67 (14)
C14—C11—N3	113.66 (12)	O5—Cl1—O3	109.73 (11)
C14—C11—H11A	108.8	O6—Cl1—O4	109.55 (12)
N3—C11—H11A	108.8	O5—Cl1—O4	109.66 (10)
C14—C11—H11B	108.8	O3—Cl1—O4	107.13 (10)
N3—C11—H11B	108.8	C18^i^—C18—C17	121.9 (2)
H11A—C11—H11B	107.7	C18^i^—C18—H18	119.1
N2—C9—C10	110.42 (13)	C17—C18—H18	119.1
N2—C9—H9A	109.6	C12—C13—C14	119.23 (15)
C10—C9—H9A	109.6	C12—C13—H13	120.4
N2—C9—H9B	109.6	C14—C13—H13	120.4
			
C6—N2—C7—C8	−178.35 (13)	C1—N1—C5—C4	0.3 (3)
C9—N2—C7—C8	60.97 (17)	C3—C4—C5—N1	0.5 (3)
C8—N3—C10—C9	−57.20 (16)	C16—C15—C14—C13	−0.8 (2)
C11—N3—C10—C9	174.41 (12)	C16—C15—C14—C11	−178.52 (15)
C10—N3—C8—C7	57.58 (16)	N3—C11—C14—C15	−101.07 (17)
C11—N3—C8—C7	−174.15 (12)	N3—C11—C14—C13	81.28 (19)
N2—C7—C8—N3	−59.62 (17)	C9—N2—C6—C3	−170.06 (14)
C1—C2—C3—C4	1.9 (3)	C7—N2—C6—C3	69.22 (18)
C1—C2—C3—C6	−174.69 (16)	C4—C3—C6—N2	28.0 (2)
C10—N3—C11—C14	59.12 (18)	C2—C3—C6—N2	−155.56 (16)
C8—N3—C11—C14	−67.06 (18)	C5—N1—C1—C2	0.1 (3)
C6—N2—C9—C10	177.63 (14)	C3—C2—C1—N1	−1.2 (3)
C7—N2—C9—C10	−60.81 (17)	C16—N4—C12—C13	0.2 (2)
N3—C10—C9—N2	59.18 (17)	O2—C17—C18—C18^i^	−18.1 (3)
C2—C3—C4—C5	−1.6 (2)	O1—C17—C18—C18^i^	160.9 (2)
C6—C3—C4—C5	174.91 (16)	N4—C12—C13—C14	−1.6 (3)
C12—N4—C16—C15	0.9 (2)	C15—C14—C13—C12	1.8 (2)
C14—C15—C16—N4	−0.6 (3)	C11—C14—C13—C12	179.51 (15)

Symmetry codes: (i) −*x*+1, −*y*+1, −*z*+1.

### Hydrogen-bond geometry (Å, °)

**Table:**

*D*—H···*A*	*D*—H	H···*A*	*D*···*A*	*D*—H···*A*
N3—H3N···N4^ii^	0.925 (19)	1.884 (19)	2.8067 (19)	175.0 (17)
O1—H1A···N1^iii^	0.93 (2)	1.68 (2)	2.6081 (19)	178 (2)
C10—H10A···O3^iv^	0.99	2.37	3.281 (2)	153 (2)
C12—H12···O6^v^	0.95	2.49	3.138 (3)	126 (2)

Symmetry codes: (ii) *x*−1, *y*, *z*; (iii) *x*, *y*+1, *z*; (iv) *x*−1, *y*+1, *z*; (v) −*x*+2, −*y*+1, −*z*.
